# Arrhythmias in children undergoing orthotopic heart transplantation

**DOI:** 10.3389/fcvm.2023.1323958

**Published:** 2024-01-11

**Authors:** Eser Doğan, Fırat Ergin, Mehmet B. Beyter, Gülçin K. Kaşıkçı, Şeyma Ş. Ön, Oğuzhan Ay, Reşit E. Levent, Çağatay Engin, Zülal Ülger

**Affiliations:** ^1^ Department of Pediatric Cardiology, Faculty of Medicine, Ege University, İzmir, Türkiye; ^2^ Department of Cardiovascular Surgery, Faculty of Medicine, Ege University, İzmir, Türkiye

**Keywords:** arrhythmia, pediatric, transplantation (heart), ECG, Holter ECG

## Abstract

**Introduction:**

Heart transplantation (HT) is the only treatment option in children with heart failure secondary to cardiomyopathies and non-reparable congenital heart diseases.

**Methods:**

We performed a retrospective clinical data review of all consecutive pediatric patients (aged 2–18 years) who underwent orthotopic HT for advanced heart failure at our institution between January 2007 and January 2023. Clinical, procedural, and follow-up data were collected and comprehensively analyzed.

**Results:**

We identified 27 children (66.7% males) with a median age of 15 years (IQR: 7–16) and a median weight of 45 kg (IQR: 22–66) at the time of the intervention. 24 patients (88.8%) were diagnosed with dilated cardiomyopathy, 2 (7.4%) with restrictive cardiomyopathy, and 1 (3.7%) with hypertrophic cardiomyopathy. On a median follow-up of 35.07 months (IQR: 13.13–111.87), arrhythmias were detected in 9 (33%) patients. Three patients developed symptomatic sinus node dysfunction at 18, 25, and 38 days and received permanent pacemakers. One patient developed a complete AV block during acute rejection at 76 months and received a temporary pacemaker. Two patients developed chronic sinus tachycardia at 4 and 16 months and were treated with Beta-blockers after eliminating all causes of sinus tachycardia. One patient developed a complete right bundle branch block at 12 months. One patient developed ventricular extrasystole at 10 months and was found to have grade 2 rejection. An Atrial extrasystole was detected in one patient at 96 months. We did not identify significant risk factors for arrhythmias post-HT.

**Discussion:**

After pediatric HT, early-onset rhythm disturbances, often attributed to surgery-related issues such as sinus node dysfunction, may necessitate invasive treatments like permanent pacemaker therapy. Close monitoring post-transplantation is crucial, and routine follow-up with Holter ECG is necessary to identify potential rhythm disorders even in the absence of symptoms. Rhythm disturbances that develop during follow-up can serve as early indicators of graft rejection and should be carefully evaluated.

## Introduction

Congestive heart failure (CHF) is a clinical syndrome caused by the heart's inability to pump enough blood to meet the metabolic needs of the body. There are marked differences in the causes and mechanisms of CHF between adults and children (1). Heart failure in children is usually secondary to serious congenital heart diseases and cardiomyopathies that cannot be corrected with medical and surgical treatments (2, 3). Heart transplantation (HT) is the best option for long-term life expectancy in children with CHF (3). HT has been practiced in pediatric patients for many years and is associated with various complications (4). However, if these complications are effectively monitored and treated, a significant life span and quality of life increase can be achieved after HT. Factors such as denervation of the transplanted heart, autonomic nervous system modulation, immunosuppression, and rejection increase the risk of arrhythmia and sudden cardiac arrest (5). Few studies evaluate the frequency of arrhythmias after HT in children (6, 7). The most common rhythm disorders include chronic sinus tachycardia, sinus bradycardia, supraventricular tachycardia, non-sustained ventricular tachycardia, and atrial extrasystoles (6, 8). Herein, we examine our institutional experience with pediatric HT and evaluate the characteristics of postoperative rhythm disorders and the potential associated risk factors.

## Methods

### Study design and patient selection

We performed a retrospective clinical data review of all consecutive pediatric patients (aged 2–18 years) who underwent orthotopic HT for advanced heart failure at our institution between January 2007 and January 2023. Clinical, procedural, and follow-up data were collected and comprehensively analyzed. Approval from the institutional review board was obtained. Written informed consent was signed by the patients or their legal guardians to perform the procedure and to use their clinical records for publication.

### Heart transplantation

The pre-transplantation clinical evaluation consisted of transthoracic echocardiography (TTE), right and left heart catheterization, respiratory function tests, and detailed specialist assessments of other systems. After this multidisciplinary evaluation, the HT council reviewed each case. The decision to proceed with listing for HT was based on an estimated life expectancy lower than 1 year. Patients were added to the transplant list according to ABO blood group, weight, and height. Hearts from donors with body weight no more than 30% below that of the recipient were considered safe (5). Heart transplantation was performed using the biatrial technique in all patients.

### Immunosuppressive therapy

Immunosuppression after HT is divided into three main categories: initiation, maintenance, and rejection treatment. The early postoperative period has a substantial risk of rejection, even if the patient is hemodynamically stable. During this period, we used short-term initiation therapy to protect against the negative effects of calcineurin inhibitors on renal functions, especially in patients with hemodynamic instability. For this purpose, we preferred polyclonal antibody rabbit anti-thymocyte globulin or interleukin-2 receptor blockers, which have fewer side effects. For maintenance treatment, we applied triple immunosuppressive therapy consisting of calcineurin inhibitors (cyclosporine or tacrolimus), antiproliferative agents (mycophenolate mofetil), and corticosteroids. In patients with intolerance to calcineurin inhibitors, we used Sirolimus and Everolimus (interleukin-2 inhibitors). Immunosuppressive therapy was started immediately before HT. 5–10 mg/kg of intravenous methylprednisolone was given before surgery and at the bypass exit. Oral Prednisone was then initiated at a dose of 1 mg/kg and then tapered. Mycophenolate mofetil was started immediately before surgery. We added calcineurin inhibitors to the treatment in the early postoperative period. We kept the doses of immunosuppressive agents high for the first 3 months, then reduced the intensity of immunosuppression.

### Follow-up

Endomyocardial biopsy was the gold standard for the diagnosis of acute rejection. Biopsies were evaluated according to ISHLT criteria (9). Biopsies were performed under general anesthesia weekly for the first month, once every 2 weeks for the next 3 months, monthly for the next 6 months, and once every three months for the following year. In the following years, an endomyocardial biopsy was performed according to the patient's clinical condition. Biopsy was performed earlier in patients with suspected rejection. Coronary artery angiography was performed annually. Two independent observers reviewed all angiograms. Allograft vasculopathy was diagnosed in case of luminal narrowing, distal tapering, or discrete stenosis. Follow-up TTE after heart transplantation was performed at 1 week, 2 weeks, and monthly thereafter. In our institution, patients were followed in the pediatric cardiology clinics up to the age of 18 and were then transferred to adult cardiology teams. Therefore, the follow-ups included in this study are till the transfer to the adult cardiology team or death.

### Holter ECG monitoring

Follow-up electrocardiogram (ECG) after HT was performed at 1 week, 2 weeks, and monthly thereafter. Holter ECG was done yearly or more frequently for patients exhibiting symptoms or arrhythmia on ECG. Holter ECG recordings were obtained over 24 h using a 6-channel Holter ECG device (DMS 300-7 Holter Reader; DMS, Stateline, NV). The traces were evaluated by experienced pediatric cardiologists using the Cardio Scan 12.0 program (DM Software, Inc.). Postoperative and follow-up arrhythmias and the corresponding treatments were noted.

### Statistical analysis

Statistical analyses were performed using SPSS Version 25.0 (IBM, Armonk, NY, USA). Categorical variables were reported as frequency and percentage and continuous variables were represented as median with interquartile range (IQR). The normality of measurements was assessed using the Shapiro**-**Wilk test. Statistical analyses were conducted using the Mann**–**Whitney *U*-test for continuous variables and by chi-square test and Fisher's exact test for categorical variables as appropriate. A *P*-value <0.05 was considered statistically significant. All reported *P* values are two-sided.

## Results

### Patient demographics

We identified 27 children (66.67% males) with a median weight of 45 kg (IQR: 22–66) and a median height of 157 cm (IQR: 123–170) at the time of the HT. The median age was 15 years (IQR: 7–16) (Table 1). Of these HT recipients, 24 (88.8%) were diagnosed with dilated cardiomyopathy, 2 (7.4%) with restrictive cardiomyopathy, and 1 (3.7%) with hypertrophic cardiomyopathy. A left ventricular assist device was implanted in 16/24 patients diagnosed with dilated cardiomyopathy before HT. The frequency of hemodynamically significant arrhythmias of atrial and ventricular origin was 33% among our transplant candidates under follow-up with ventricular assist devices. At the presentation, one patient had concomitant chronic renal failure, one had diabetes, and one had Becker muscular dystrophy. The patient with renal failure also underwent kidney transplantation in our center.

**Table 1:** Recipient demographics, procedural data, and post-transplant outcomes.

Parameter	Recipient patients (*n* = 27)	No arrhythmia (*n* = 18)	Follow-up arrhythmia (*n* = 9)	*P* value
Weight (kg), median (IQR)	45 (22–66)	48.5 (23.75–61.75)	45 (22–66)	0.898^a^
Height (cm), median (IQR)	157 (123–170)	159.5 (128–170.75)	155 (123–167)	0.662^a^
Body surface area (m^2^), median (IQR)	1.3 (1.2–1.42)	1.3 (1.2–1.39)	1.25 (1.2–1.45)	0.847^a^
Age at surgery (years), median (IQR)	15 (7–16)	14 (7.75–16.25)	15 (6.5–16.5)	0.917^a^
Preoperative Tricuspid annular plane systolic excursion (mm), median (IQR)	17.5 (17–18)	17.5 (17–18.25)	18 (17–18.5)	0.750^a^
Transpulmonary gradient mean, median (IQR)	8 (7–9)	8 (7–8.25)	7 (7–10.5)	0.937^a^
Mean pulmonary artery pressure (mmHg), median (IQR)	31 (28–34)	32 (28–34.75)	30 (28–33.5)	0.515^a^
LVAD implantation, *N* (%)	16	9 (56.25)	7 (43.75)	0.167^b^
Graft ischemia time (min), median (IQR)	149 (87–225)	153 (88.75–240.75)	145 (96–214.5)	0.662^a^
Cardiopulmonary bypass time(min), median (IQR)	160 (96–255)	188.5 (103.75–245.5)	155 (95–215)	0.858^a^
Aortic cross-clamp time (min), median (IQR)	108 (75.75–128.25)	108 (79.75–127.25)	102 (75–130)	0.990^a^
ICU stay (days), median (IQR)	6 (5–8)	6 (5.75–8)	5 (4.5–6)	0.181^a^
Follow-up time (months), median (IQR)	35.07 (13.13–11.87)	31.33 (8.4–81.3)	50.54 (19.5–128.8)	0.194^a^
Follow-up LVEF (%), median (IQR)	58 (57–64)	60 (57–65.5)	60 (56.5–63)	0.820^a^
Rejection, *N* (%)	3	1 (33)	2 (66)	–
Donor/recipient weight ratio, median (IQR)	1.28 (1.2–1.35)	1.30 (1.22–1.34)	1.27 (1.23–1.36)	0.571^a^

LVAD, left ventricular assist device; ICU, intensive care unit; LVEF, left ventricular ejection fraction.

^a^ Mann–Whitney *U*-test.

^b^ Fisher's exact test.

### Follow-up

Immunosuppressive therapy was initiated in all patients as described in the methods. An endomyocardial biopsy was performed according to protocol. Grade 2 rejection was detected in 3 patients and regressed with immunosuppressive therapy. One patient had a single coronary ostium anomaly in the transplanted heart. No other coronary anomaly was detected on follow-up coronary angiograms. There was no coronary artery vasculopathy during follow-up.

### Rhythm disturbances

The patients underwent a median of 12 (IQR: 7–18) ECGs and a median of 3 (IQR: 2–5) 24-h Holter ECGs. Arrhythmias were detected in 9 (33.33%) patients during follow-up. The demographic findings, TTE parameters, and post-transplant rhythm disorders of patients who developed follow- up arrhythmias are summarized in Table 2. Three patients developed symptomatic sinus node dysfunction at 18, 25, and 38 days and received permanent pacemakers. In two of these patients, sinus node dysfunction was believed to be related to the surgical technique used. In the other case, the donor's heart had a long ischemia time, and the recipient was hemodynamically unstable and on extracorporeal membrane oxygenation support. One patient developed a complete atrioventricular block due to rejection at 76 months of follow-up and was treated with a temporary pacemaker. The heart block regressed after 72 h, and the sinus rhythm resumed. This patient's ventricular ejection fraction was 40% during rejection but normalized after adjusting their immunosuppressive therapy. Persistent sinus tachycardia was detected in two patients at the 4 and 16 months of follow-up. Beta-blockers were initiated after eliminating all plausible causes of sinus tachycardia. Their heart rates remained within normal limits for their age. Atrial and ventricular extrasystole were each detected in one patient. The incidence of atrial extrasystoles was less than 5% and no episodes of supraventricular tachycardia attacks were observed. The ventricular extrasystole rate remained below 5% and no instances of bigeminy, couplets, or ventricular tachycardia were observed. The patient with ventricular extrasystole was found to have grade 2 rejection. The immunosuppressive treatment was revised and a response was obtained. These two patients were asymptomatic with had low-frequency arrhythmias and were thereby followed clinically. One patient developed a complete right bundle branch block at 12 months with no hemodynamic changes.

**Table 2:** Details of the pediatric patients who developed arrhythmias after heart transplantation.

	Patient 1	Patient 2	Patient 3	Patient 4	Patient 5	Patient 6	Patient 7	Patient 8	Patient 9
Age (years)	17	16	10	6	17	15	15	7	6
Sex	Male	Male	Male	Male	Male	Female	Male	Male	Female
Weight (kg)	66	67	42	19	70	50	45	22	23
Height (cm)	165	170	140	116	175	167	155	123	125
Primary diagnosis	Dilated CMP	Dilated CMP	Dilated CMP	Dilated CMP	Dilated CMP	Dilated CMP	Dilated CMP	Dilated CMP	Restrictive CMP
Arrhythmia type	Sinus node dysfunction	Ventricular extrasystole	Sinus node dysfunction	Sinus node dysfunction	Chronic sinus tachycardia	Chronic sinus tachycardia	Complete right bundle branch block	Transient complete AV block	Atrial extrasystole
Medical treatment	–	–	–	–	Beta-blocker	Beta-blocker	–	–	–
Pacemaker implantation	+	–	+	+	–	–	–	+ Temporary	–
Previous LVAD	+	+	+	+	+	+	+	–	–
Complications	–	–	Constrictive pericarditis	–	–	–	–	Acute kidney failure	–
Follow-up time (months)	13.6	25.6	113.3	146	13.13	40.13	50.53	111.86	144.46
Rejection	–	Grade 2	–	–	–	–	–	Grade 2	–
Death	–	–	+ Constrictive pericarditis	–	–	–	–	–	–

LVAD, left ventricular assist device; CMP, cardiomyopathy; AV, atrioventricular.

Except for the patients with rejection and complete heart block, none of the patients who developed arrhythmia had low ventricular ejection fraction. No electrophysiological study was required during the study period. The 5-year survival rate of all patients was 82%. In the group in which arrhythmia was detected during follow-up, one patient with sinus node dysfunction and a permanent pacemaker died secondary to constrictive pericarditis. The other two patients with permanent pacemakers are monitored without any complications. The surviving 8 patients with postoperative arrhythmias are monitored with no change in their medical treatments.

There was no significant difference in mortality between patients with or without arrhythmias. Patients' age, height, weight, pre-transplant ventricular assist device implantation, follow-up time, cardiac biopsy results, and procedural parameters were assessed as risk factors for post-transplant arrhythmia, and no significant results were observed (Table 1).

## Discussion

Orthotopic HT remains the most effective treatment method for patients with end-stage CHF. Because of the scarcity of heart donors, the number of ventricular assist device implantations is steadily increasing. The 5-year survival rate after HT is 75%–80%, which is still higher than that with ventricular assist devices (10). In our series, 82% of the patients survived at least 5 years, consistent with the literature.

Various arrhythmias that can occur after HT and their associated causes have been described (9). Depending on the surgical technique, atrial arrhythmias originating from the suture site sinus node dysfunction, and complete heart block due to damage to the sinus node can be seen with the biatrial and bicaval techniques. Prolonged graft ischemia may be associated with secondary progressive conduction system disorders and postoperative atrial fibrillation. Surgical autonomic denervation of the transplanted heart can result in an elevated resting heart rate, elevated heart rate response to exercise, atrial fibrillation, or sudden cardiac death.

Hyperacute rejection can cause atrial fibrillation and ventricular tachycardia. Chronic rejection can cause atrial fibrillation and accompanying left ventricular dysfunction and ventricular tachycardia. Coronary artery vasculopathy can lead to progressive conduction system disorders, atrial fibrillation, and ventricular tachycardia. Graft failure may cause atrial and ventricular tachycardia, complete heart block, and sudden cardiac death (9). In adult studies, the most common arrhythmias are reentrant arrhythmias related to the anastomosis line (11). In pediatric studies, common arrhythmias include chronic sinus tachycardia, sinus bradycardia, supraventricular tachycardia, non-sustained ventricular tachycardia, and atrial extrasystoles (6). We observed similar rhythm disorders in our series. Three patients developed sinus node dysfunction postoperatively and received permanent pacemakers. Permanent pacemaker implantation after HT has been reported in adults (10, 12). Heart transplant patients are known to be denervated, which results in a higher resting heart rate, usually between 90 and 110 beats per min. Several disease states have demonstrated that sinus tachycardia results in poor long-term outcomes in patients with severe heart failure and severe coronary artery disease (13). Only 2 patients with chronic sinus tachycardia were started on beta-blocker therapy. The right bundle branch block is a common electrocardiographic abnormality in heart transplant recipients, but the cause remains unknown (14).

Studies have shown that patients who develop arrhythmia after HT have higher rates of rejection, coronary artery disease, and mortality (15). However, we observed no statistically significant difference in mortality according to the presence of arrhythmia in our patients. Those who developed sinus node dysfunction in the early postoperative period underwent permanent pacemaker**s** before discharge. Apart from sinus node dysfunction, other rhythm disorders that developed during follow-up were not found to cause rapid hemodynamic decompensation.

Patients undergo a multidisciplinary evaluation in our clinic before HT. In children, patients alongside their caregivers are evaluated carefully in terms of treatment adherence. Patients have the greatest adherence to follow-up and treatment after HT. Increased experience with HT and improvement in the immunosuppression regimens and imaging techniques also reduce the complication rates (16). Grade 2 rejection was detected in three endomyocardial biopsies performed during routine follow-up. One of those patients developed a complete heart block, one developed ventricular extrasystole, and no arrhythmia was detected in the other patient. These uncommon rhythm disorders support the increased risk of arrhythmia associated with heart transplant rejection (17). Although endomyocardial biopsies were performed, right and left heart catheterization was also done to assess for coronary artery diseases, and no coronary artery disease was detected. In the absence of acute graft rejection, electrophysiologic studies may elucidate the substrate of atrial arrhythmias.

Electrical cardioversion and radiofrequency ablation may be considered in a heart transplant recipient with symptomatic and persistent atrial arrhythmias (18). Atrioventricular re-entry tachycardia (AVRT) or atrioventricular nodal re-entry tachycardia (AVNRT) is uncommon and related to an underlying electrophysiological substrate within the donor heart (19). Symptomatic AVRT and AVNRT can be managed with radiofrequency ablation of the accessory pathway and slow pathway, respectively. The sinus tachycardias that developed in our patient group responded to beta-blocker treatment and thus there was no need for electrophysiological studies.

Sudden death is a well-recognized mode of death in heart transplant recipients. The variable incidence of reported sudden death is probably due to the small sample sizes of existing studies, heterogeneity in the populations studied, and inconsistent or unspecified definitions used. Acute rejection and coronary artery disease are major risk factors for sudden death in adult heart transplant recipients (20). Clinicians should consider endomyocardial biopsy and coronary angiography in HT recipients with atrial or ventricular arrhythmias to exclude acute rejection and assess for coronary artery disease (20). Grade 2 rejection was detected in the biopsy of our patient with ventricular extrasystole, which supports this theory.

### Limitations

This study includes a relatively small number of patients. However, the rhythm disorders and their treatments detected in our study support the high number of adult studies in literature. Larger pediatric studies are needed.

## Conclusions

After pediatric HT, early-onset rhythm disturbances, often attributed to surgery-related issues such as sinus node dysfunction, may necessitate invasive treatments like permanent pacemakers. Close monitoring post-HT is crucial, and routine follow-up with Holter ECG is necessary to identify rhythm disorders even in the absence of symptoms. Follow-up rhythm disturbances can serve as early indicators of rejection.

## Data Availability

The raw data supporting the conclusions of this article will be made available by the authors, without undue reservation.
